# Diagnostic Utility of Hysteroscopic Biopsy in Cases of Suspected Lobular Endocervical Glandular Hyperplasia and Comparison with Cervical Conization

**DOI:** 10.3390/healthcare11111619

**Published:** 2023-06-01

**Authors:** Reona Shiro, Yasushi Kotani, Mamiko Ohta, Hanako Sato, Yoko Kashima, Kosuke Murakami, Kaoru Kawasaki, Hidekatsu Nakai, Noriomi Matsumura

**Affiliations:** Department of Obstetrics and Gynecology, Kindai University Faculty of Medicine, Osakasayama 589-8511, Japan; reonashiro@med.kindai.ac.jp (R.S.); nakai@med.kindai.ac.jp (H.N.);

**Keywords:** lobular endocervical glandular hyperplasia, conization, hysteroscopic biopsy

## Abstract

Background: Cervical cystic lesions encompass a range of benign and malignant pathologies. Magnetic resonance imaging or cytology alone cannot provide a definitive diagnosis, and conventional practice involves performing a cervical biopsy by conization to confirm the histology in cases exhibiting potential signs of lobular endocervical glandular hyperplasia (LEGH) or malignancy. However, as postoperative complications resulting from conization can impact future fertility and pregnancy, alternative diagnostic methods are needed for reproductive-age patients. This study aimed to establish the efficacy of a hysteroscopic biopsy for diagnosing cervical cystic lesions and compare it with conization. Methods: Thirteen patients with cervical cystic lesions suspected of LEGH or malignancy underwent a hysteroscopic biopsy, while 23 underwent conization. Patient background information, preoperative evaluation, histology, and postoperative outcomes were collected and compared retrospectively. Results: No significant differences were found between the hysteroscopy and conization groups in terms of mean patient age (45 vs. 48 years), operating time (23 vs. 35 min), blood loss (small amount vs. 43 mL), and postoperative hospitalization (1.1 vs. 1.6 days). Conclusion: A hysteroscopic biopsy allows for targeted resection of the cervix while maintaining diagnostic accuracy. It may serve as an efficient method for diagnosing cervical cystic lesions.

## 1. Introduction

Cystic lesions of the uterine cervix encompass a range of benign and malignant conditions, including nabothian cysts, lobular endocervical glandular hyperplasia (LEGH), and gastric-type mucinous carcinoma, including minimal deviation adenocarcinoma (MDA) [1]. LEGH, first described by Nucci et al., is a lesion that needs to be differentiated from MDA and is considered a potential precursor to cervical cancer [2,3,4]. The incidence of LEGH is rare, estimated at 0.7% [5]. It commonly affects women in their 40 s and 50 s presenting with typical symptoms such as increased vaginal discharge. However, some cases are asymptomatic, posing a challenge for early detection [6]. Ultrasonography, a standard tool in obstetric and gynecological examinations, is insufficient for a definitive diagnosis. In cases where cervical polycysts are observed on an ultrasound, magnetic resonance imaging (MRI) is often performed.

Characteristic imaging findings, such as the “cosmos pattern”, have been reported for LEGH and other malignant lesions [7,8]. Although a MRI is expected to aid in the diagnosis, a definitive diagnosis cannot rely solely on imaging [9,10]. Another suggested diagnostic method involves the detection of gastric mucin by collecting cervical mucus and using a HIK1083 latex agglutination kit [11]. However, this method often fails to achieve successful detection, rendering it an adjunct to the diagnosis [12]. While cervical cytology is commonly performed in gynecological practices and can identify atypical glandular cells in some cases of LEGH and malignancy, other cases may yield a diagnosis negative for intraepithelial lesions or malignancy. Therefore, when MRI findings of patients with cervical cystic lesions suggest the possibility of LEGH or malignancy, a cervical biopsy of the cystic lesions and histopathological assessment, including immunostaining, are required to confirm the current conditions [2,5].

Traditionally, cervical biopsy by conization has been the standard approach for cervical cystic lesions [7]. However, in such cases, cervical conization often results in a deeper resection of the cervix compared to the conventional conical resection performed for conditions such as cervical intraepithelial neoplasia (CIN). This is due to the frequent proximity of cystic lesions to the internal os of the uterus. Deeper cervical resection increases the risk of miscarriage and preterm birth due to cervical shortening during postoperative pregnancy [13,14]. Moreover, it raises the risk of postoperative cervical stenosis and closure, potentially reducing fertility in women of childbearing age [15]. Although cervical conization is considered a fertility-preserving procedure for reproductive-age females, it can inadvertently complicate future fertility and pregnancies. Deep conization for a biopsy site can negatively impact the quality of life in older patients, as cervical stenosis and closure can lead to menstrual abnormalities and hematometra [16]. Therefore, concerns arise regarding the long-term complications associated with deep conization for biopsy purposes.

To date, no literature has been published regarding the postoperative complications of cervical conization or the risk of miscarriage and preterm birth in cases of cystic lesions of the uterine cervix requiring a cervical biopsy for diagnosis. However, it is widely recognized that a history of deeper resection through cervical conization for CIN cases increases the risk of miscarriage and preterm delivery [17,18], suggesting a similar outcome for cervical cystic lesions. Even if a deeper resection of the cervix is performed in cases suspected of LEGH or malignancy, the biopsy may not yield reliable histological findings to determine whether uterine preservation is a viable option for the patient. Consequently, some patients may opt for a total hysterectomy due to concerns about overlooking malignancy. This decision presents a significant challenge for individuals desiring pregnancy, as they may face permanent infertility if no abnormalities are found, leading to an irreversible loss of fertility.

Alternative methods for diagnosing cervical cystic lesions that are less invasive yet maintain diagnostic accuracy are crucial, particularly for patients of reproductive age. One such approach involves the use of a hysteroscope for the biopsy. A hysteroscope is an endoscope that is inserted through the cervix into the uterine corpus [19]. Originally designed for submucosal fibroids and endometrial polyps protruding into the uterine cavity, hysteroscopic surgery allows for resectioning the lesion using various types of electrodes located at the tip of the hysteroscope. The excised tissue is then removed from the uterus through the cervical canal [20,21,22]. This technique provides surgeons with direct visualization of the cystic lesions from within the uterus, allowing for targeted biopsy and minimizing the extent of resection while ensuring an accurate diagnosis.

While a cervical biopsy of cystic lesions using transcervical resection with a hysteroscope has been reported previously [8], the limited number of cases hinders the ability to establish the effectiveness of a hysteroscopic biopsy. In our study, we performed hysteroscopic biopsies on 13 cases, with MRI findings indicating the potential presence of LEGH or malignancy. This report compares these cases and previous cases that underwent conventional conization for biopsy purposes.

## 2. Materials and Methods

**Figure 1:**
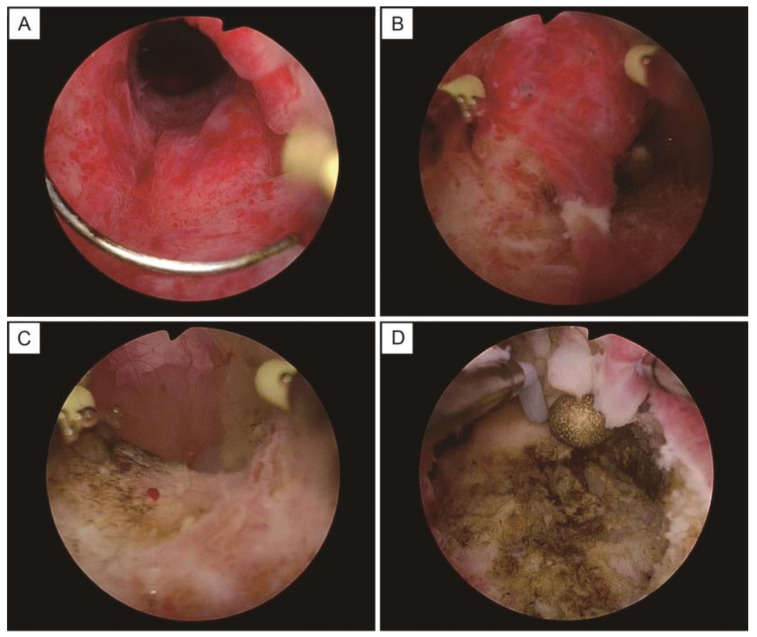
The images of a hysteroscopic biopsy. (**A**) The lesions are detected as elevated cysts around the cervical canal. (**B**) The surfaces of cysts are excised with a loop electrode. (**C**) The inner cavities of the cysts are exposed, with the mucinous contents leaking out of the cysts. (**D**) The bleeding sites are coagulated with a ball electrode.

Patients referred to Kindai University Hospital for multiple cystic lesions in the cervix underwent pelvic MRI scans. Those whose T2-weighted images demonstrated the cosmos pattern (diffuse or microcystic parts surrounded by medium-to-large cysts) [8], suggesting LEGH or malignancy, were offered a transcervical biopsy by hysteroscopic resection. The patients who agreed to a hysteroscopic biopsy received cervical dilation through the transcervical insertion of a laminaria tent the day before the biopsy. Transcervical resection using monopolar was performed using the OES Pro resectoscope (OLYMPUS Co., Tokyo, Japan), equipped with the HF surgery system UES-40S (OLYMPUS Co., Tokyo, Japan), a high-frequency power generator with an output power of 50 W for excision and 50 W for coagulation. The scope was fitted with a loop electrode for excision and a ball electrode for coagulation. During the hysteroscopic procedure, 3% D-sorbitol was perfused. The hysteroscopic observation revealed elevated cysts around the cervical canal (Figure 1A). As the surfaces of the cysts were resected, the inner cavities were exposed, and mucinous contents leaked out (Figure 1B,C). The cyst surface was removed around the entire circumference of the cervical canal. Bleeding areas were coagulated, and the hysteroscope was removed from the uterus once hemostasis was confirmed (Figure 1D). Patients were discharged from the hospital on the first postoperative day and scheduled for a follow-up appointment at the outpatient clinic in 2–4 weeks.

Second, we reviewed similar cases of cervical cystic lesions over the past decade at our hospital, which required cervical conization for a biopsy. Cervical resection was performed in a conical shape using HARMONIC SYNERGY^®^ (Johnson & Johnson, Tokyo, Japan), an ultrasonic scalpel, for the conization procedure. A hooked blade was attached to the device for resection. Since the lesions were typically located near the internal os of the uterus, resection was performed with a depth of 2 cm or more to ensure complete removal of the lesions. Patients were discharged on the first postoperative day.

In this study, we assessed 13 patients who underwent a hysteroscopic biopsy and 23 patients who underwent conventional conization when a preoperative diagnosis of LEGH or higher lesions was suspected but not confirmed. Conization was the chosen technique for both procedures between 2011 and 2019. Conversely, a hysteroscopic biopsy was performed on the cases between 2020 and 2022. Therefore, it is important to note that the two groups in this study are not contemporaneous but rather represent a retrospective study with different study periods.

Patient information such as backgrounds, preoperative assessment, perioperative complications, and postoperative outcomes was collected for each group from their medical charts. The diagnostic accuracy of the pathology results was also evaluated in patients who underwent a hysteroscopic biopsy or conization followed by total hysterectomy. Additional details regarding the histological diagnosis were examined for cases involving a hysterectomy. A comparison was made between the two groups in terms of the mean age of the patients, operation time, blood loss, and postoperative hospital stay. Statistical analysis was performed using a Student’s *t*-test, with a significance level set at *p* < 0.05. This study received approval from the institutional review board of the Kindai University Faculty of Medicine (RR03-25).

## 3. Results

Thirteen patients underwent a hysteroscopic biopsy for cervical cystic lesions between April 2020 and September 2022. Table 1 presents the patients’ backgrounds, preoperative assessments, and postoperative outcomes. Of the 13 patients who had a hysteroscopic biopsy, five had nabothian cysts, and three had no significant histological findings. Two cases were diagnosed with LEGH, while one case revealed gastric-type mucinous carcinoma. All patients diagnosed with LEGH and mucinous carcinoma underwent a total hysterectomy. In the case of a LEGH diagnosis, two patients were given the options of a hysterectomy or observation with close monitoring, and both chose the former. One patient opted for a hysterectomy to mitigate the potential risk of developing cervical cancer, while the other desired treatment to alleviate abundant mucinous discharge.

**Figure 2:**
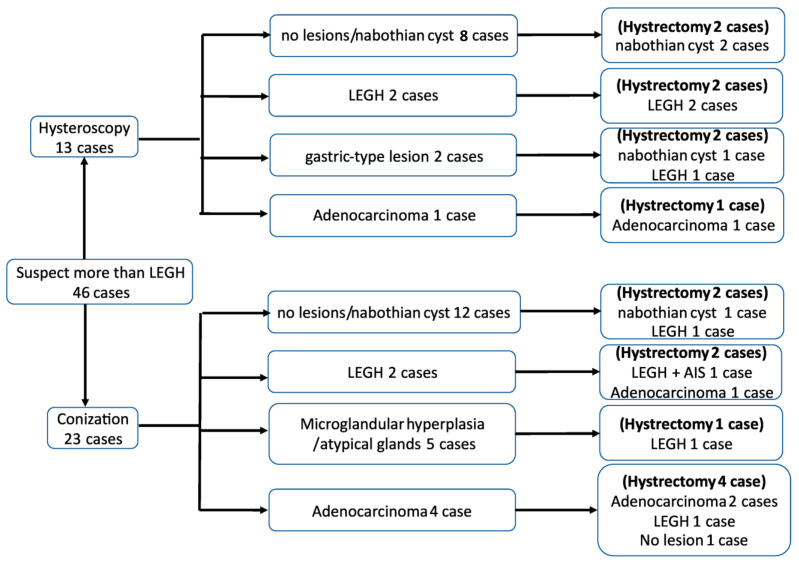
Flowchart of the postoperative pathology results for hysteroscopy surgery and conization. Abbreviations: LEGH: lobular endocervical glandular hyperplasia and AIS: adenocarcinoma in situ.

From January 2011 to March 2020, a total of 23 patients with cervical cystic lesions underwent cervical conization for a biopsy. The patients’ backgrounds, preoperative assessments, and postoperative outcomes are detailed in Table 2. Among the cases, 12 were diagnosed with nabothian cysts or showed no significant histological findings. LEGH was identified in two cases, while malignancy was detected in four cases (three cases of MDA and one case of endocervical mucinous carcinoma). Among the cases that underwent a total hysterectomy after conization, two cases initially diagnosed with LEGH had additional findings of AIS and mucinous carcinoma. Despite two cases being diagnosed with no findings and glandular hyperplasia, a recommendation was made to proceed with a hysterectomy following conization due to concerns that the procedure might have failed to reach the lesions, resulting in a potential underdiagnosis. A flowchart illustrating the postoperative pathology results for each technique is presented in Figure 2.

In principle, a hysteroscopic biopsy was performed in cases of suspected LEGH or higher, and the diagnostic accuracy is shown in Table 3 for cases in which a total hysterectomy was finally performed. The sensitivity was 100%, the specificity was 67%, the positive predictive value was 80%, and the negative predictive value was 100%. Table 4 shows the results of the same study for conization. The sensitivity was 86%, specificity 50%, positive predictive value 86%, and negative predictive value 50%.

A comparison between the hysteroscopy and conization groups revealed no significant difference in the mean age of the patients (45 vs. 48 years). The hysteroscopy group had a relatively shorter mean operative time (23 vs. 35 min), although the difference was not statistically significant. Minimal blood loss was observed in all hysteroscopy cases, with an average of 43 mL in the conization procedures. The postoperative hospital stays were 1.1 and 1.6 days, respectively, with no significant difference observed (Table 5).

## 4. Discussion

**Figure 3:**
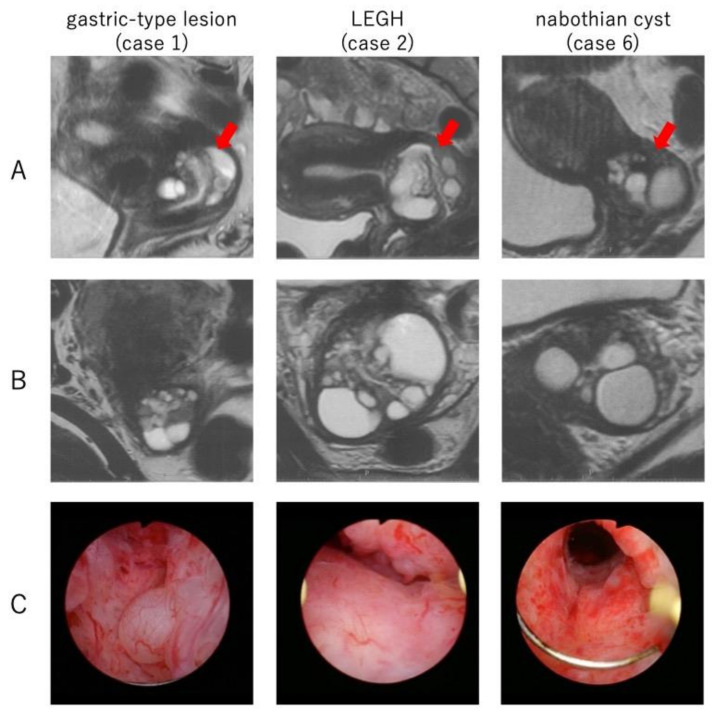
MRI of the uterine cervix, and images of the lesions by hysteroscopic observation. (**A**) T2-weighted image, sagittal section. Cystic lesions in the cervix are stretching to the internal os of the uterus (arrows). (**B**) T2-weighted image, horizontal section. The sizes of the lesions and cysts differ among cases. (**C**) Cervical cystic lesions observed from inside the cervix by a hysteroscope.

We have used both hysteroscopic resection and conization as biopsy methods for cervical cystic lesions, with the MRI findings indicating the potential for LEGH or malignancy. The MRI findings in this study revealed a range of lesion sizes or cysts within the lesions, which were consistently identified as elevated cysts during hysteroscopy (Figure 3). More than half of the patients were diagnosed with nabothian cysts or no lesions. In cases of LEGH, which carry an inherent risk of developing into malignancy [6], a total hysterectomy could be considered for patients not seeking pregnancy, while close observation with cytology and an imaging assessment should be recommended for those desiring pregnancy [23,24,25]. However, two cases in the conization group were diagnosed with cervical malignancy post-hysterectomy, despite the preoperative biopsy indicating LEGH. Therefore, the possibility of underdiagnosing becomes a concern when conization is used for a biopsy with a result of LEGH. Conversely, two cases in the hysteroscopy group initially diagnosed with gastric-type lesions were ultimately found to have nabothian cysts and LEGH, respectively, after a hysterectomy. Consequently, determining the need for additional interventions based on this diagnosis has become a matter for further consideration.

Conization is a commonly employed biopsy method for cervical cystic lesions. However, compared to conization performed for CIN, it necessitates a deeper resection, which can result in increased blood loss and a longer operative time. Furthermore, it is associated with obstetric complications, such as a 1.5- to 3-times higher risk of preterm delivery during pregnancy compared to the overall rate of 8 to 15% [13]. Recent reports have also demonstrated a correlation between deep conical resection and an elevated risk of preterm delivery [26]. Complications arising from cervical conization include cervical stenosis and cervical atresia [15], which may require additional intervention [27,28]. In severe cases, a total hysterectomy may be the only viable option [28]. Therefore, attempts are being made to develop devices that can be inserted into the cervical canal [27,28]. Nonetheless, it is crucial to avoid complications such as cervical canal stenosis or closure.

Hysteroscopic procedures are associated with specific complications, including uterine perforation and fluid overload [29,30,31]. However, since the operative time was typically under half an hour in most cases, the risk of fluid overload due to a prolonged operative time was unlikely to be a concern. In our previous cases, we utilized a monopolar technique without an electrolyte solution. Nevertheless, in recent years, the mainstream approach has shifted towards a bipolar biopsy with an electrolyte solution. As a result, we now perform bipolar hysteroscopic biopsies in our department. Our protocol recommends excising only the surfaces of the lesions that can be visualized as elevated cysts around the cervical canal using a hysteroscope. There is no need for deep cervical resection to access the lesions, thereby minimizing the risk of uterine perforation. Although there may be concerns about the adequacy of tissue collection, the fact that the hysteroscopic biopsy in this study allowed for a pathological assessment without compromising the diagnostic yield supports the effectiveness of our protocol. Therefore, the excision technique used in hysteroscopic biopsies minimizes the loss of normal cervical tissue. While there were no significant differences in the perioperative complications between hysteroscopic resection and conization, the hysteroscopy group exhibited shorter operative times and minimal blood loss. Conversely, conization was associated with an increased operative time and blood loss in a few cases, likely due to the necessity for deep conization, which carries a higher risk of bleeding. Even when deep resection was performed during conization, two cases in the conization group eventually required a hysterectomy due to concerns about an underdiagnosis. Hysteroscopic resection allows surgeons to visualize cystic lesions and confirm the biopsy site, ensuring the quality of the biopsy. It is considered an alternative method for efficiently and minimally invasively conducting biopsies of cervical cystic lesions.

Regarding the costs of both procedures, in Japan, a conization surgery costs the patient approximately 33,300 JPY (about 247 USD) in terms of surgical expenses (additional fees for anesthesia and other services apply). The disposable ultrasonic scalpel HARMONIC SYNERGY^®^ costs 47,000 JPY (about 348 USD) at the listed price (the actual delivered price is slightly lower). On the other hand, the cost of a monopolar hysteroscopic procedure is 47,300 JPY (about 350 USD), while the bipolar procedure using saline solution costs the patient approximately 66,300 JPY (about 491 USD). Regarding the equipment, both loop and roller electrodes have a listed price of 15,000 JPY (about 111 USD). However, there is a potential for cost reduction for these electrodes. For saline bipolar, both loop and roller electrodes have a listed price of 19,000 JPY (about 141 USD). These electrodes are disposable and must be discarded after use. Various techniques can be used for both conization and hysteroscopic surgery, each with its associated costs. However, a cost comparison conducted at our hospital revealed no significant difference between the two procedures (note that hysteroscopic surgery requires an initial investment in equipment such as monitors).

One limitation of this study is the small number of cases and the short duration of follow-up. While the long-term complications resulting from cervical conization have been extensively discussed, the impact of partial resection of the cervix through a hysteroscopic approach on the cervical physiology has not been well documented. In this series of cases, the postoperative effects of hysteroscopic resection on fertility and future pregnancies remain unclear, as no patients have attempted pregnancy following the hysteroscopic biopsy. Since hysteroscopic resection involves minimal cervical resection compared to deep conization, it is anticipated to carry a lower risk for fertility and future pregnancies. Further investigation and data accumulation are necessary to assess the long-term complications associated with this biopsy method. Additionally, this study focuses on the examination of the technique itself. The most crucial aspect of a new technique is diagnostic accuracy. We believe that the accuracy of this new method should be reevaluated as the number of cases increases in the future. Nevertheless, the current data are comparable to those obtained through conventional methods.

## 5. Conclusions

A hysteroscopic biopsy holds promise as a valuable method for diagnosing cystic lesions of the cervix. The key advantage of this technique lies in direct visualization of the lesion through an endoscope, enabling targeted biopsy of the identified area. However, further studies are required to expand the number of cases, extend the observation period, and investigate additional aspects such as diagnostic accuracy.

## Figures and Tables

**Table 1:** Patients’ background, preoperative evaluation, postoperative diagnosis, and postoperative course of cases in the hysteroscopy group.

Case	Age	GP	Gastric Mucin	Cervical Cytology	Pathology after Biopsy	Hysterectomy after Biopy	Surgery	Pathology after Hysterectomy
1	44	G0	N/A	LSIL	gastric-type lesion	Y	TLH	nabothian cyst
2	45	G4P4	positive	NILM	LEGH	Y	TLH	LEGH
3	33	G0	N/A	AGC-FN	gastric-type mucinous carcinoma	Y	LRH	gastric-type mucinous carcinoma, pT2a2
4	31	G0	negative	NILM	nabothian cyst	N		
5	50	G3P3	negative	NILM	nabothian cyst	N		
6	37	G1P0	positive	NILM	gastric-type lesion	Y	TLH	LEGH
7	48	G1P1	N/A	AGC-FN	nabothian cyst	N		
8	48	G2P2	negative	NILM	no lesions	Y (request by the patient)	TLH	nabothian cyst
9	59	G4P3	negative	NILM	nabothian cyst	N		
10	49	G1P1	N/A	NILM	nabothian cyst	Y (due to hypermenorrhea)	TLH	nabothian cyst
11	44	G2P2	negative	NILM	no lesions	N		
12	48	G4P1	N/A	AGC-NOS	LEGH	Y	TLH	LEGH
13	49	G0	N/A	AGC-NOS	no lesions	N		

Abbreviations: GP: gravida/para, N/A: not applicable, LSIL: low-grade squamous intraepithelial lesion, NILM: negative for intraepithelial lesion or malignancy, AGC-FN: atypical glandular cells—favor neoplastic, AGC-NOS: atypical glandular cells—not otherwise specified, LEGH: lobular endocervical glandular hyperplasia, Y: Yes, N: No, TLH: total laparoscopic hysterectomy, and LRH: laparoscopic radical hysterectomy.

**Table 2:** Patients’ background, preoperative evaluation, postoperative diagnosis, and postoperative course of cases in the conization group.

Case	Age	GP	Gastric Mucin	Cervical Cytology	Pathology after Biopsy	Hysterectomy after Biopsy	Surgery	Pathology after Hysterectomy
1	37	G0	N/A	NILM	no lesion	N		
2	40	G0	N/A	NILM	no lesion	N		
3	55	G6P3	N/A	NILM	nabothian cyst	N		
4	41	G2P2	N/A	NILM	nabothian cyst	N		
5	59	G1P1	N/A	AGC	nabothian cyst	N		
6	35	G1P1	N/A	NILM	no lesion	N		
7	33	G0	N/A	NILM	nabothian cyst	Y (due to hypermenorrhea)	TAH	nabothian cyst
8	69	G1P0	N/A	AGC	no lesion	N		
9	31	G0	N/A	AGC	MDA	Y	mRH	MDA
10	48	G1P0	N/A	AGC	MDA	Y	RH	LEGH
11	46	G1P1	N/A	AGC	LEGH	Y	TLH	LEGH, AIS
12	46	G2P1	N/A	AGC	microglandular hyperplasia	N		
13	81	G4P3	N/A	adenocarcinoma	nabothian cyst	N		
14	44	G0	N/A	AGC-NOS	no lesion	Y	TLH	LEGH
15	55	G4P3	N/A	NILM	LEGH	Y	TAH	mucinous carcinoma
16	45	G0	N/A	NILM	microglandular hyperplasia	N		
17	42	G1P1	N/A	AGC-NOS	nabothian cyst, microglandular hyperplasia	N		
18	44	G2P1	N/A	NILM	microglandular hyperplasia	Y	TLH	LEGH
19	46	G0	N/A	AGC	MDA	Y	RH	LEGH
20	37	G0	N/A	NILM	atypical glands	N		
21	56	G4P3	N/A	AGC	endocervical adenocarcinoma, usual type	Y	TLH	no lesion
22	52	G3P2	N/A	ASC-US	CIN 1, nabothian cyst	N		
23	51	G2P2	N/A	NILM	no lesion	N		

Abbreviations: GP: gravida/para, N/A: not applicable, NILM: negative for intraepithelial lesion or malignancy, AGC: atypical glandular cells, AGC-NOS: atypical glandular cells—not otherwise specified, ASC-US: atypical squamous cells of undetermined significance, MDA: minimal deviation adenocarcinoma, LEGH: lobular endocervical glandular hyperplasia, CIN: cervical intraepithelial neoplasia, Y: Yes, N: No, TAH: total abdominal hysterectomy, mRH: modified radical hysterectomy, TLH: total laparoscopic hysterectomy, RH: radical hysterectomy, and AIS: adenocarcinoma in situ.

**Table 3:** Diagnostic accuracy in the hysteroscopic biopsy and total hysterectomy.

		Total Hysterectomy		
		LEGH or Greater Lesion	Nabothian Cyst No Lesion	Total
Hysteroscopic Biopsy	LEGH or greater lesion	4	1	5
	nabothian cyst no lesion	0	2	2
	Total	4	3	7

**Table 4:** Diagnostic accuracy in the conization and total hysterectomy.

		Total Hysterectomy		
		LEGH or Greater Lesion	Nabothian Cyst No Lesion	Total
Conization	LEGH or greater lesion	6	1	7
	nabothian cyst no lesion	1	1	2
	Total	7	2	9

**Table 5:** Comparison of patients’ backgrounds and surgical outcomes between a hysteroscopic biopsy and a cervical conization.

	Hysteroscpopy (*n* = 13)	Conization (*n* = 23)	*p* Value
mean age (year)	45 ± 8	48 ± 12	0.49
operative time (minute)	23 ± 12	35 ± 20	0.06
total blood loss (mL)	small amounts (not countable)	43 ± 70	
postoperative hospital stay (day)	1.1 ± 0.2	1.6 ± 1.4	0.22

## Data Availability

Not applicable.
